# Prevalence of pain-free weeks in chiropractic subjects with low back pain - a longitudinal study using data gathered with text messages

**DOI:** 10.1186/2045-709X-19-28

**Published:** 2011-12-14

**Authors:** Nadège Lemeunier, Alice Kongsted, Iben Axén

**Affiliations:** 1 Institut Franco-Européen de Chiropratique, Toulouse, France; 2 Nordic Institute of Chiropractic and Clinical Biomechanics, Odense, Denmark; 3 Unit of Intervention and Implementation Research, Institute of Environmental Medicine, The Karolinska Institutet, Stockholm, Sweden

## Abstract

**Introduction:**

The use of automated text messages has made it possible to identify different courses of low back pain (LBP), and it has been observed that pain often fluctuates and that absolute recovery is rather rare. The purpose of this study was to describe the prevalence of pain-free weeks and pain-free periods in subjects with non-specific LBP treated by chiropractors, and to compare subjects from two different countries in these aspects.

**Methods:**

Data were obtained from two practice-based multicentre prospective outcome studies, one Danish and one Swedish, involving subjects being treated by chiropractors for non-specific LBP. Over 18 weeks, subjects answered a weekly automated text message question on the number of days in the past week that they had experienced bothersome LBP, i.e. a number between 0 and 7. The number of weeks in a row without any LBP at all ("zero weeks") as well as the maximum number of zero weeks in a row was determined for each individual. Comparisons were made between the two study samples. Estimates are presented as percentages with 95% confidence intervals.

**Results:**

In the Danish and the Swedish populations respectively, 93/110 (85%) and 233/262 (89%) of the subjects were eligible for analysis. In both groups, zero weeks were rather rare and were most commonly (in 40% of the zero weeks) reported as a single isolated week. The prevalence of pain free periods, i.e. reporting a maximum of 0, 1 or 2, or 3-6 zero weeks in a row, were similar in the two populations (20-31%). Smaller percentages were reported for ≥ 7 zero weeks in a row. There were no significant differences between the two study groups.

**Conclusion:**

It was uncommon that chiropractic subjects treated for non-specific LBP experienced an entire week without any LBP at all over 18 weeks. When this occurred, it was most commonly reported for brief periods only. Hence, recovery in the sense that patients become absolutely pain free is rare, even in a primary care population.

## Introduction

Low back pain (LBP) is a very common condition with an annual prevalence of at least 50% [1]. However, approximately 80% of patients suffering from LBP are diagnosed with non-specific LBP [2] because the specific causes are rather rare. One consequence is that it is difficult to find an effective treatment for this large, heterogeneous group of patients, which probably explains the limited treatment effect generally obtained in scientific trials[3,4].

Traditionally, randomized clinical trials and outcome studies have the disadvantage of measuring outcome at only a few points in time, leaving an absence of data for the periods in between. However, for patients the time "in between" is as important as some arbitrarily selected points in time, such as at 6 or 12 months after treatment. Further, LBP often runs an episodic or fluctuating course [5-8] which is ignored by follow ups measured only at one or a few points in time. Commonly, the proportion of subjects who are improved at these time points is estimated. This approach fails to take the trajectory of pain into account, thereby not fully describing recovery or improvement. With the use of diaries or automated text messages it is possible to follow the course of a disease more closely, whether treated or not.

In this study, text messaging was used to record weekly follow-ups over a period of 18 weeks in two populations of chiropractic subjects with LBP, one in Denmark and another in Sweden. By means of these data, it was possible to identify individual clinical courses of LBP. It was confirmed that pain often fluctuated [9,10] and that, although the majority of subjects improved to some degree, absolute recovery was rather rare [6,11]. Presumably, this is contrary to what clinicians in primary care expect, namely a gradual more or less stable path of improvement. The expectations of clinicians have been found to be considerably higher than the patient-reported outcomes in a quality assurance study of a secondary care spine centre [12]. Obviously, if the care-giver and the patient have higher expectations of the outcome than what they will experience, both will be dissatisfied. For patients, this may result in a lengthy period of doctor shopping. For the clinician, it may result in a prolonged treatment course, because unrealistic effects are aimed for. More evidence concerning course and recovery patterns of non-specific LBP is therefore needed [13]. The frequently collected data from the two mentioned LBP studies [6,11] made it possible to study the frequency with which chiropractic subjects experienced pain free periods over a stretch of time as well as the length of such periods. The purpose of this study was to perform secondary analyses of these data in order to describe the prevalence of pain-free weeks and periods in subjects with non-specific LBP treated by chiropractors, and to compare two populations of different nationalities in these aspects. In the primary analyses, the aim was to describe the clinical course without focus on pain-free weeks.

Knowledge of this type will make it possible for practitioners to inform patients about the most likely evolution of his/her LBP problem. By analysing prospective data from different populations and countries, wider generalisations regarding clinical outcome would be possible.

## Methods

Data were obtained from two practice-based multicentre prospective outcome studies involving subjects being treated by chiropractors for non-specific LBP; one was conducted in Denmark and one in Sweden. Both studies have been reported elsewhere [6,9-11].

Prior to inclusion, subjects received written and verbal information about the study. The projects were presented to the relevant local ethics committees. In Denmark, the committee stated that the study did not need approval. In Sweden, approval was granted by the local ethics committee at the Karolinska Institutet (2007/1458-31/4).

### Study populations

Seven chiropractors were invited to participate in the Danish study on the condition that they followed an instruction program on how to examine and diagnose their patients according to an evidence-based classification system [9,14]. Subjects aged 18-65 years with non-specific LBP, who had not had chiropractic treatment for the present episode of LBP prior to the consultation in the present clinic, were consecutively included (n = 110).

In the Swedish study, 35 chiropractors who had been compliant in a previous practice-based study were recruited. These chiropractors were found to be similar to the members of the Swedish Chiropractors' Association (In Swedish: Legitimerade Kiropraktorers Riksorganisation) regarding age, gender and years in practice [11]. Altogether, 262 subjects with non-specific LBP were enrolled. The age range of these subjects was 16-69 years and they had not been under chiropractic care for the past 3 months.

In both populations, subjects were considered to have non-specific LBP as cases of significant pathologies would have been referred out. Other exclusion or non-inclusion criteria were: previous back surgery (Danish study), pregnancy, and inability to read Danish/Swedish. Not having a mobile phone, not knowing how to use the text message function or not wanting to participate in the study obviously also precluded participation in the studies.

The gender distribution was fairly similar in the two studies with 45% and 48% females, respectively [9,11]. Most subjects had LBP only, but 46% in the Danish and 50% in the Swedish study had also leg pain. Twenty five per cent of the Danish study participants had experienced LBP for more than 3 months and 57% of the Swedish participants reported to have had LBP for more than 30 days in the past year. Further detailed information concerning LBP duration was not collected at baseline.

Six of the seven Danish chiropractors had graduated from the University of Southern Denmark whereas the majority (56%) of the Swedish chiropractors had graduated from a chiropractic college in England (Anglo-European College of Chiropractic in Bournemouth) and a further 13% from the University of Southern Denmark. The remaining Swedish chiropractors had an American or Australian chiropractic degree.

Previous studies of chiropractors in the Scandinavian countries have shown that their treatment will consist of spinal manipulative therapy (SMT), mobilization, soft tissue treatment, massage, advice, exercise therapy and information [15-17]. The SMT can be either purely manual or assisted by mechanical devices (drop-piece tables and activators). The appropriate treatment was decided by the chiropractors in the two studies and not taken into account in our analyses.

Previous comparisons between drop-outs and participants in the two original studies revealed that the drop-outs in the Danish study were more likely to be men, to have leg pain and to report short duration LBP [9]. In the Swedish study, however, gender and leg pain were not significantly different between drop-outs and those who stayed in the study, but the Swedish drop-outs had a shorter duration of LBP in the past year and reported lower pain intensity [10].

### Clinical procedures, data collection and validity of data

In the Danish study, the chiropractors collected baseline data using a standardized physical examination protocol [14], as described in detail elsewhere [6], treated "as usual" and followed every week over 18 weeks with the help of automated text messages, using the SMS-Track-Q program [18]. Eighteen weeks follow- up was chosen for practical reasons. The data collection took place from February till October 2008.

In the Swedish study, after recording base-line data, the clinical examination and treatment were decided by the chiropractors according to usual practice. Subjects were followed by weekly text-messages using the same data collection method as in Denmark but over 6 months[11]. In the present study, to allow comparisons with the Danish study, only data from the first 18 weeks have been included. The data collection took place from May 2008 to June 2009.

Thus in both studies subjects received a weekly automated text message question concerning the number of days in the past week that they had 1) been bothered by LBP (Danish study) or 2) experienced bothersome LBP (Swedish study). The subjects replied via a text message with a number between 0 and 7. If no reply message had been received within 4 days, a reminder was automatically sent. If no reply had been received when the next week's text message question was sent, the data was recorded as "missing".

The text message questions were:

In Denmark: "Using a number from 0 to 7, please answer how many days you have been bothered by your low back pain this week." In Sweden: "How many days during this previous week has your low back pain been bothersome (i.e. affected your daily activities or routines?). Please answer by a number from 0 to 7."

A thorough scrutiny of the SMS-Track method using data from the Swedish study indicated that the SMS-Track system yields high response rates not affected by season, is user friendly and has good compliance [10]. An inter-reliability study showed the SMS-Track method to be superior to retrospective data collection in subjects with non-specific LBP [19]. A comparison between pain intensity (none/moderate/severe) and number of days with pain per week revealed almost identical profiles of these two variables over 18 weeks in the Danish study [9]. The outcome measure used herein, "zero weeks", i.e. the weeks a subject reports zero days of bothersome LBP, is therefore assumed to describe the absence of pain.

### Data analysis

The weekly text message replies were automatically transferred into a data file to be used for analysis. The resulting spread sheets were analysed by hand in the following manner:

• First, each row (representing one subject) was checked for number of weeks without a response, "missing" data. Subjects who had missing data for altogether 11 weeks or more were arbitrarily defined as below a minimally satisfactory compliance level. They were excluded from the analyses because it was considered impossible to obtain a truthful impression of their clinical course. The remaining missing values were considered to represent at least one day with LBP in order to evaluate a "worst case" scenario.

• Second, the number of weeks in a row without pain ("zero weeks") was counted during the 18 weeks in each of the two populations. Thus, every zero week was classified in relation to the number of zero weeks in a row, regardless if several such periods were found in the same individual. For ease of presentation, these results were then reduced into six categories: 1, 2, 3, 4, 5-9, and 10-18 weeks in a row with no LBP.

• Third, the maximum number of zero weeks in a row per subject was counted and grouped into one of five categories: 0, 1 or 2, 3-6, 7-10, or 11-18 weeks in a row with no LBP. This means that only one value, the maximum number of zero weeks, was calculated for each individual, with possible values from 0 to 18.

• Finally, two additional analyses were performed. First, a more generous definition of no pain was used, allowing for 1 or 2 days of pain in a week also to be counted as a zero week ("best case") in subjects originally included in the analysis. Second, no subjects were withdrawn from the analysis and all missing data were simply interpreted as not being zero-weeks ("worst case"). A best and a worst case scenario have been chosen to illuminate the effect of: on the one hand, keeping to a strict definition of no pain, and on the other hand, keeping to a strict definition of compliance.

Results are displayed as bar graphs including 95% confidence intervals (CIs). The two study samples were compared and differences between estimates considered statistically significant when the CIs did not overlap.

## Results

### Participants

**Figure 1:**
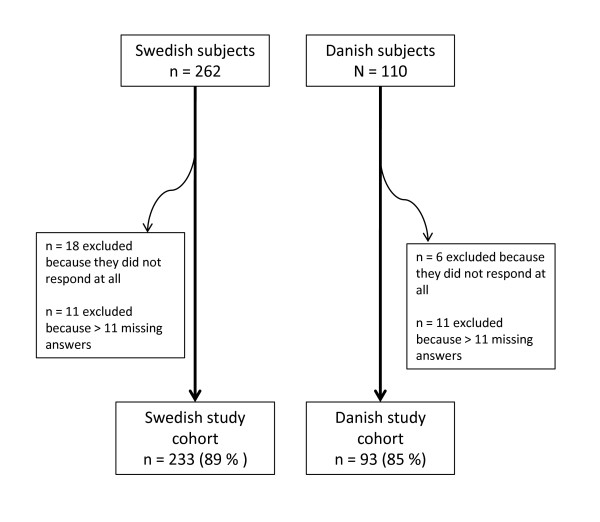
**Flowchart showing the number of participants in two clinical outcome studies**.

Seventeen individuals from the Danish study were excluded because they had more than 11 missing answers. The corresponding number in the Swedish study was 29 (Figure 1). The number of subjects with valid data was therefore 93 (85%) and 233 (89%) respectively, in the final Danish and Swedish data files.

### Missing data in the two study populations

**Figure 2:**
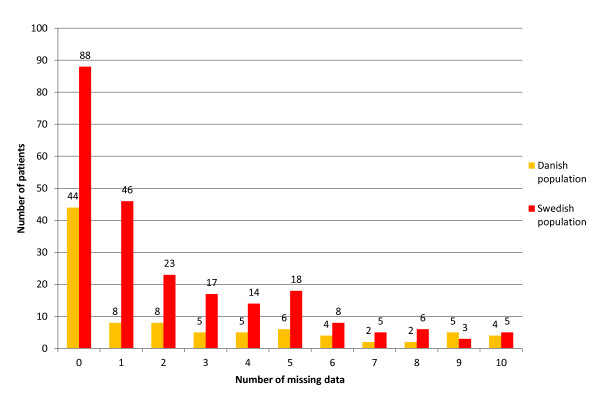
**Frequency distribution of missing data**. The numbers of patients are shown for the Danish and the Swedish populations separately.

In the two populations, 49 Danish patients (53%) and 145 Swedish patients (32%) had some missing data. The frequency distribution of the missing data is reported in Figure 2. In both populations, the number of missing data rarely exceeded 9 for any individual.

### Number of "zero weeks" in a row in the two populations

**Figure 3:**
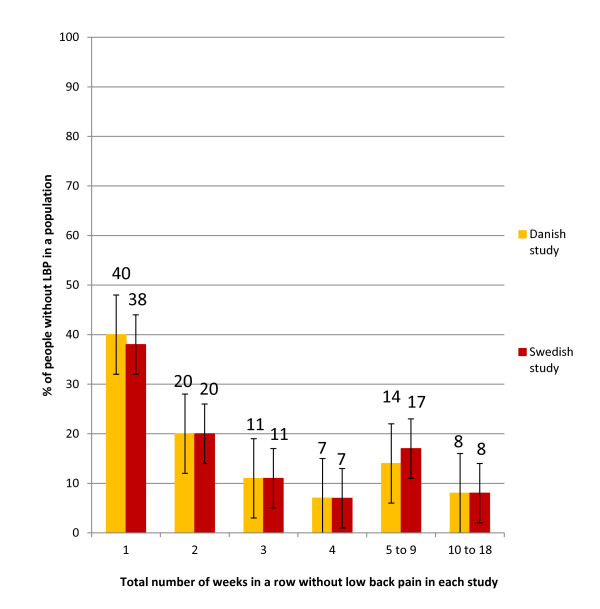
**Number of weeks in a row with 0 days of low back pain for 326 subjects in two clinical outcome studies**. The percentages are shown for the Danish and Swedish populations separately. Bars indicate the 95% confidence intervals.

Throughout the study period, the reported weekly number of days with LBP was rarely zero. When a zero week was present, it appeared as a single week in about 40% of cases (Figure 3). The proportion of subjects with more than one zero week in a row diminished as the number of weeks in a row increased (Figure 3). Almost identical results were found in the two populations and the 95% CIs always overlapped (Figure 3).

### Maximum number of "zero weeks" in a row per subject in the two populations

**Figure 4:**
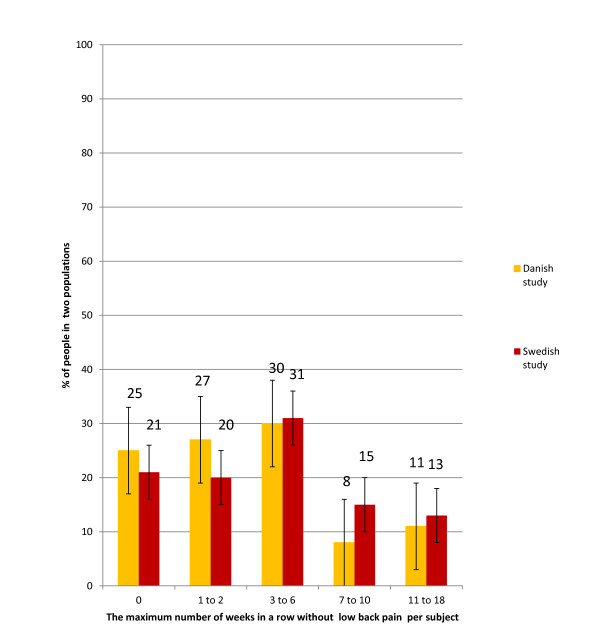
**Maximum number of weeks in a row without low back pain per subject in two clinical outcome studies**. The percentages are shown for the Danish and Swedish populations separately. Bars indicate the 95% confidence intervals.

As can be seen in Figure 4, the proportions of subjects reporting a maximum of 0, 1 or 2, or 3-6 zero weeks in a row were similar in the two populations (20-31%) regardless category. Smaller percentages were reported for 7-10 weeks (8% and 15% in the two cohorts, respectively), and for 11-18 zero weeks in a row (11% and 13%, respectively) (Figure 4). There were no statistically significant differences between the two study groups, as the 95% CIs overlapped.

### Additional analyses

The two additional analyses did not alter the picture revealed in the original results. This was true both when allowing for a more generous definition of the absence of pain, namely also 1 or 2 days of LBP in the week and when assuming that all missing data represented weeks with LBP (data available on request).

## Discussion

### Summary of findings

This study showed that weeks with no LBP days at all were rare during an 18 week period following a first visit to a chiropractor. Most often, the subjects reported to have had at least one day with LBP in any given week. Furthermore, having a pain free week most commonly occurred on its own as an isolated event. In fact, one third and one fourth of subjects, respectively, in the Danish and Swedish studies, never experienced a single zero week during 18 weeks. Despite the fact that most patients were expected to improve fairly quickly [6,11] very few reported being absolutely pain free for any longer periods. This finding raises the question whether the pain free episodes can be predicted from baseline variables. However, this was not one of our aims but should be investigated in future studies.

### Methodological considerations

As this was not a randomized study with an untreated control group, it is not possible to determine whether there were any associations between the therapeutic approach and the observed course of LBP. The results observed here may therefore have occurred as a result of treatment but may also merely reflect the natural course. However, it is reasonable to assume that each chiropractor did his/her best at tailoring the treatment to the needs of each subject in order to obtain an optimal result and that the outcome truthfully reflects what generally happens in daily clinical practice. No similar studies were found in the literature for comparison.

In all, 15% and 11% of the original Danish and Swedish participants were not included in our analysis due to missing data. Further, as is often the case, previous analyses had demonstrated some differences between participants and drop-outs [6,10], and revealed that the drop-outs tended to report short duration of LBP [9].This would possibly lead to an underestimation of pain-free weeks. However, the number of drop-outs in each population was very low (11 and 15%), thus rendering this risk minimal. We consider the final study samples fairly representative of the typical chiropractic patient with non-specific LBP, due to the nature of the studies and the high participation rates.

These analyses were performed according to the worst case principle, in the sense that missing data were never interpreted as a zero week, not even if an isolated week with missing data was surrounded by one or several zero weeks. This approach ensures that subjects did at least as well as our result suggests. Obviously, missing data could be both LBP and no LBP, but we chose this approach instead of imputing data for missing values, since a majority (53% and 62% respectively) of the included subjects had some missing values. However, the missing data did not alter the results as the majority of the respondents only had 1 to 5 weeks of missing data, and few patients had more than 5 weeks of missing data (17/93 and 27/233 respectively in the Danish and the Swedish populations).

In the additional analysis, the findings were similar regardless definition of zero week. A more lenient definition of "LBP free", including both 1 and 2 days of pain in one week did not change the results. This indicates that LBP, when present, usually consist of more than the occasional pain. A more "severe" interpretation of data did not influence our findings either, indicating that our results provide a good picture of reality.

### Limitations of gathering data with SMS

Frequent monitoring of patients with LBP using a few simple questions can obviously provide information on clinical course. The text message method is, however, not a suitable technique to obtain more detailed data on the state of the LBP problem. Indeed, only limited amounts of data can be sent in a text message. Therefore, by choosing the term "bothersomeness", which has been found to be associated with pain intensity, disability and psychological health [20], it is believed that the relevant clinical features of the LBP condition is covered in one single question.

### Comparison of the two study populations

The fact that the results were similar between the two cohorts, even though the questions to the subjects were somewhat differently worded in the two studies, strengthens the validity of these findings.

The similar results in the two studies might seem surprising as the involved chiropractors worked differently. The Danish chiropractors had been selected on quite special criteria and had to put considerable effort into the clinical examination of their patients with the purpose of obtaining an evidence-based diagnosis. This may have resulted in a selection of a more academically minded, well-informed and skilled type of clinician. It is also possible that their evidence-based diagnoses would have resulted in a more effective treatment and therefore more zero weeks. Also the Swedish chiropractors had been selected on some criteria of "excellence", as they had been compliant in a previous practice-based study, and therefore perhaps were typical of more competent clinicians. Their examination and diagnostic procedures had not, however, been "streamlined" in any fashion. Therefore we suspected that they might produce less favourable results than their Danish colleagues, but this could not be demonstrated from the simple outcome "zero weeks". Possibly, other outcomes could have revealed some differences between the cohorts.

## Conclusion

It was uncommon that chiropractic subjects with non-specific LBP experienced an entire week without bothersome LBP during a course of 18 weeks. When this occurred, it was most commonly reported for brief periods only. Hence, recovery in the short term, in the sense that patients become absolutely pain free for longer periods, is rare, even in a primary care population.

### Recommendations

• To learn more about the patterns of the clinical and the natural courses of non-specific LBP, it would be relevant to study this phenomenon for a range of patient populations, in the general population and for different types of treatment.

• Moreover, these results emphasize the importance of measuring recovery over a time-span rather that at one given point. This applies both to researchers and to clinicians who want to undertake quality assurance projects in their own clinic.

• It is essential that these findings are taken into account by teaching institutions in order to prepare students for the clinical reality already at the undergraduate level.

## Competing interests

The authors declare that they have no competing interests.

## Authors' contributions

NL was responsible for the analysis of data and participated in the manuscript preparation. AK was responsible for the design of the Danish study and the supervision of data collection. IA was responsible for the design of the Swedish study and the supervision of data collection. AK and IA were supervising the study process and were involved in the manuscript preparation. All the authors revised and approved the final manuscript.

## Author's information

1: Institut Franco-Européen de Chiropratique, Toulouse, France

2: Nordic Institute of Chiropractic and Clinical Biomechanics, Odense, Denmark

3: Unit of Intervention and Implementation Research, Institute of Environmental Medicine, The Karolinska Institutet, Stockholm, Sweden
